# Kinetic Features of L,D-Transpeptidase Inactivation Critical for β-Lactam Antibacterial Activity

**DOI:** 10.1371/journal.pone.0067831

**Published:** 2013-07-04

**Authors:** Sébastien Triboulet, Vincent Dubée, Lauriane Lecoq, Catherine Bougault, Jean-Luc Mainardi, Louis B. Rice, Mélanie Ethève-Quelquejeu, Laurent Gutmann, Arul Marie, Lionel Dubost, Jean-Emmanuel Hugonnet, Jean-Pierre Simorre, Michel Arthur

**Affiliations:** 1 Centre de Recherche des Cordeliers, Equipe 12, Université Pierre et Marie Curie–Paris 6, UMR S 872, Paris, France; 2 INSERM, U872, Paris, France; 3 Université Paris Descartes, Sorbonne Paris Cité, UMR S 872, Paris, France; 4 CEA, DSV, Institut de Biologie Structurale (IBS), Grenoble, France; 5 CNRS, UMR 5075, Grenoble, France; 6 Université Joseph Fourier, Grenoble 1, France; 7 Assistance Publique-Hôpitaux de Paris, Hôpital Européen Georges Pompidou, Paris, France; 8 Rhode Island Hospital, Brown University, Providence, Rhode Island; 9 Laboratoire de Chimie et de Biochimie Pharmacologiques et Toxicologiques, Université Paris Descartes, UMR 8601, Paris, France; 10 CNRS, UMR 8601, Paris, France; 11 Muséum National d’Histoire Naturelle, USM0502, Plateforme de Spectrométrie de Masse et de Protéomique du Muséum, Paris, France; 12 CNRS, UMR8041, Paris, France; Institut Pasteur Paris, France

## Abstract

Active-site serine D,D-transpeptidases belonging to the penicillin-binding protein family (PBPs) have been considered for a long time as essential for peptidoglycan cross-linking in all bacteria. However, bypass of the PBPs by an L,D-transpeptidase (Ldt_fm_) conveys high-level resistance to β-lactams of the penam class in *Enterococcus faecium* with a minimal inhibitory concentration (MIC) of ampicillin >2,000 µg/ml. Unexpectedly, Ldt_fm_ does not confer resistance to β-lactams of the carbapenem class (imipenem MIC = 0.5 µg/ml) whereas cephems display residual activity (ceftriaxone MIC = 128 µg/ml). Mass spectrometry, fluorescence kinetics, and NMR chemical shift perturbation experiments were performed to explore the basis for this specificity and identify β-lactam features that are critical for efficient L,D-transpeptidase inactivation. We show that imipenem, ceftriaxone, and ampicillin acylate Ldt_fm_ by formation of a thioester bond between the active-site cysteine and the β-lactam-ring carbonyl. However, slow acylation and slow acylenzyme hydrolysis resulted in partial Ldt_fm_ inactivation by ampicillin and ceftriaxone. For ampicillin, Ldt_fm_ acylation was followed by rupture of the C^5^–C^6^ bond of the β-lactam ring and formation of a secondary acylenzyme prone to hydrolysis. The saturable step of the catalytic cycle was the reversible formation of a tetrahedral intermediate (oxyanion) without significant accumulation of a non-covalent complex. In agreement, a derivative of Ldt_fm_ blocked in acylation bound ertapenem (a carbapenem), ceftriaxone, and ampicillin with similar low affinities. Thus, oxyanion and acylenzyme stabilization are both critical for rapid L,D-transpeptidase inactivation and antibacterial activity. These results pave the way for optimization of the β-lactam scaffold for L,D-transpeptidase-inactivation.

## Introduction

Biosynthesis of peptidoglycan, the major constituent of bacterial cell-walls, is a preeminent target for antibiotics since the polymer is present and essential in nearly all bacterial species, with the exception of a few obligate intracellular parasites. Penicillin is the first antibiotic introduced for chemotherapy of bacterial infections and members of this drug family, the β-lactams, have remained the most broadly prescribed first-line treatment for systemic infections. The mode of action of β-lactams involves irreversible inactivation of D,D-transpeptidases, also referred to as penicillin-binding proteins (PBPs), that catalyze the last cross-linking step of peptidoglycan synthesis. The reaction links together glycan chains made of alternate β-1–4-linked *N*-acetylglucosamine (GlcNAc) and *N*-acetylmuramic acid (MurNAc) by formation of amide bonds between short peptides, which are carried by MurNAc residues from adjacent glycan chains [1]. Specifically, D,D-transpeptidases cleave the D-Ala^4^–D-Ala^5^ peptide bond of a pentapeptide stem, hence the D,D designation, and link the carbonyl of the penultimate residue (D-Ala^4^) to the side chain amine carried by the third residue of an adjacent stem peptide, thereby generating 4→3 cross-links [2]. Transpeptidation proceeds through formation of an ester bond between the catalytic serine of D,D-transpeptidases and the carbonyl of D-Ala^4^ from the acyl donor.

β-lactams are structural analogues of the D-Ala^4^–D-Ala^5^ extremity of peptidoglycan precursors and act as suicide substrates since the D,D-transpeptidases catalyze formation of an ester bond between their active-site serine and the β-lactam-ring carbonyl [3]. The resulting acylenzyme is stable leading to long-term enzyme inactivation and antibacterial activity [1]. In contrast, the ester bond connecting the active-site serine to the carbonyl of D-Ala^4^ in the physiological substrate is readily attacked by the amine of the acyl acceptor substrate resulting in 4→3 cross-link formation and enzyme turnover.

Serine-active D,D-transpeptidases have long been considered as essential for peptidoglycan cross-linking [4]. However, these enzymes can be bypassed by an unrelated enzyme family, the active-site cysteine L,D-transpeptidases (Ldts), in β-lactam-resistant mutants of *Enterococcus faecium* selected in vitro [5]. L,D-transpeptidases were subsequently identified as the main peptidoglycan cross-linking enzymes in wild-type strains of *Mycobacterium tuberculosis* [6], *Mycobacterium abscessus* [7], and *Clostridium difficile* [8]. The enzymes generate 3→3 cross-links as they cleave the peptide bond connecting the 3^rd^ and 4^th^ residues of the acyl donor and link the carbonyl of the 3^rd^ residue to the acceptor [9].

Classical PBPs and L,D-transpeptidases (Ldts) use different acyl donor substrates (stem pentapeptide versus tetrapeptide, respectively) and cleave peptide bonds of D–D and L–D configurations (D-Ala^4^–D-Ala^5^ versus the L-Lys^3^–D-Ala^4^, respectively) [9]. These differences were initially proposed to account for the lack of inhibition of *E*. *faecium* L,D-transpeptidase by ampicillin because of the aforementioned structural analogy between the β-lactam scaffold and the D-Ala^4^–D-Ala^5^ extremity of peptidoglycan precursors [5]. However, this explanation has been challenged by further analyses that unexpectedly revealed in vitro inactivation of Ldt_fm_ by β-lactams of the carbapenem class such as imipenem [10]. Activation of the L,D-transpeptidation pathway in *E*. *faecium* resulted in high-level resistance to β-lactams of the penam class, with a minimal inhibitory concentration of ampicillin (MIC) greater than 2,000 µg/ml. In contrast, carbapenems are active at low concentrations (imipenem MIC = 0.5 µg/ml), whereas β-lactams of the cephem class have a low residual activity (ceftriaxone MIC = 128 µg/ml). The molecular basis for this >4,000-fold difference in antibiotic activity is not understood. Here we have developed novel assays to investigate the lack of significant inhibition of Ldt_fm_ by penams. Several possibilities have been envisaged including low affinity for the drug, slow acylation, and hydrolysis of the acylenzyme that can account, alone or in combination, for inefficient target inactivation.

## Materials and Methods

### Chemicals

Imipenem was a gift from Merck. Ceftriaxone and ampicillin were purchased from Teva and Euromedex, respectively.

### Production and Purification of Ldt_fm_

We have previously described the construction of a derivative of vector pET2818 encoding domains I and II of Ldt_fm_ (residues 216 to 466) fused to a C-terminal 6-histidine Tag (GSH_6_) [11]. Since pET2818 encodes a β-lactamase, the insert encoding recombinant Ldt_fm_ was subcloned into vector pET28a, which confers kanamycin resistance. Ldt_fm_ was produced in *Escherichia coli* BL21 and purified by metal-affinity and size-exclusion chromatographies as previously described [12] except for the presence of kanamycin (50 µg/ml) in the culture medium. Protein concentration was determined by the Bradford method (Biorad Protein Assay) with BSA as a standard.

In order to investigate the affinity of Ldt_fm_ for β–lactams by NMR, we used a recombinant protein of smaller size, which only consisted in the catalytic domain of Ldt_fm_, and harbored a substitution of catalytic Cys by Ala in order to block acylation. Briefly, the pET28a derivative used for protein production encoded an N-terminal polyhistidine tag followed by a TEV protease cleavage site (MHHHHHHENLYFQGHM) fused to residues 341 to 466 of Ldt_fm_. Oligonucleotides 5′-ACCCGCGGTTCACACGGCGCCATCAACACCCCACCAAG-3′ and 5′-CTTGGTGGGGTGTTGATGGCGCCGTGTGAACCGCGGGT-3′ were used to introduce a Cys to Ala substitution at position 442 by site-directed mutagenesis. The protein was produced and purified as described above except that bacteria were grown in M9 minimal media containing (^13^C)glucose and ^15^NH_4_Cl. The purified protein was cleaved with 6His-labeled TEV protease. The polyhistidine tag (MHHHHHHENLYFQ) and the TEV protease were removed using NiNTA affinity resin generating recombinant enzyme consisting of residues GHM fused to residues 341 to 466 of Ldt_fm_.

### Spectrophotometry

Kinetics were performed at 20°C with a stopped-flow apparatus RX-2000 (Applied Photophysics) coupled to a Cary 100 spectrophotometer (Varian SA) in 100 mM sodium phosphate (pH 6.0). The variation in the molar extinction coefficient resulting from opening of the β-lactam ring of imipenem (−7,100 M^−1^ cm^−1^ at 299 nm), ceftriaxone (−9,600 M^−1^ cm^−1^ at 265 nm), and ampicillin (−700 M^−1^ cm^−1^ at 240 nm) were determined after alkaline hydrolysis (imipenem and ampicillin) or enzymatic hydrolysis with *Mycobacterium abscessus* β-lactamase.

### Spectrofluorometry

Fluorescence kinetic data were acquired with a stopped-flow apparatus (RX-2000, Applied Biophysics) coupled to a spectrofluorometer (Cary Eclipse; Varian) in 100 mM sodium phosphate (pH 6.0) at 20°C. The Trp residues were excited at 224 nm with a 5 nm slit and a 2 mm optical path length. Fluorescence emission was determined at 335 nm with a 5 nm slit and a 10 mm optical path length.

### Mass Spectrometry Analyses

The formation of drug-enzyme adducts was tested by incubating Ldt_fm_ with β-lactams at 20°C in water. Five microliters of acetonitrile and 1 µl of 1% formic acid were extemporaneously added, and the reaction mixture was injected directly into the mass spectrometer (Qstar Pulsar I; Applied Biosystem) at a flow rate of 0.05 ml/min (acetonitrile, 50%, water, 49.5%, and formic acid, 0.5%; per volume). Spectra were acquired in the positive mode as previously described [10].

### NMR Titrations

Increasing molar ratios of ertapenem, ampicillin, and ceftriaxone (up to 2,016, 2,058, and 1,025 equivalents, respectively) were added to a 150 µM solution of ^15^N- and ^13^C-labeled Ldt_fm_ C442A catalytic domain in 100 mM sodium phosphate (pH 6.4) containing 300 mM NaCl. Chemical shift perturbations (CSPs) were monitored at 25°C through the comparison of 2D [^1^H,^15^N]-HSQC spectra recorded at 600 MHz proton frequency and were calculated using the equation 1,
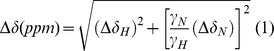
where Δ*δ_H_* and Δ*δ_N_* are the variations of chemical shifts in the proton and nitrogen dimensions, respectively, and *γ_H_* and *γ_N_* are the gyromagnetic ratio of these two nuclei. CSPs were then analyzed to extract structural and thermodynamics binding information. Peaks showing chemical shift changes greater than 0.03 ppm after addition of *ca*. 500 molar equivalents of each antibiotic were simultaneously used to calculate a dissociation constant (*K*_D_) that could be obtained from a non-linear least-square fit with equation 2,



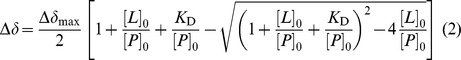
where Δ*δ* is the chemical shift perturbation at each titration point, Δ*δ*_max_ is the chemical shift difference between the free and bound forms of the protein, and [*P*]_0_ and [*L*]_0_ are the total concentrations of protein and ligand, respectively. Error on the CSP (dΔ*δ*) were estimated using equation 3,



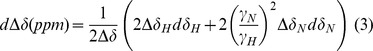
where d*δ_H_* and d*δ_N_* are the estimated absolute values of the errors committed on the determination of chemical shifts in the ^1^H and ^15^N dimensions, respectively (here d*δ_H_* = 0.005 ppm and d*δ_N_* = 0.02 ppm).

## Results

### Ldt_fm_ Displays Similar Low Affinity for β-lactams of the Carbapenem (Imipenem and Ertapenem), Cephem (Ceftriaxone), and Penam (Ampicillin) Classes

**Figure 1:**
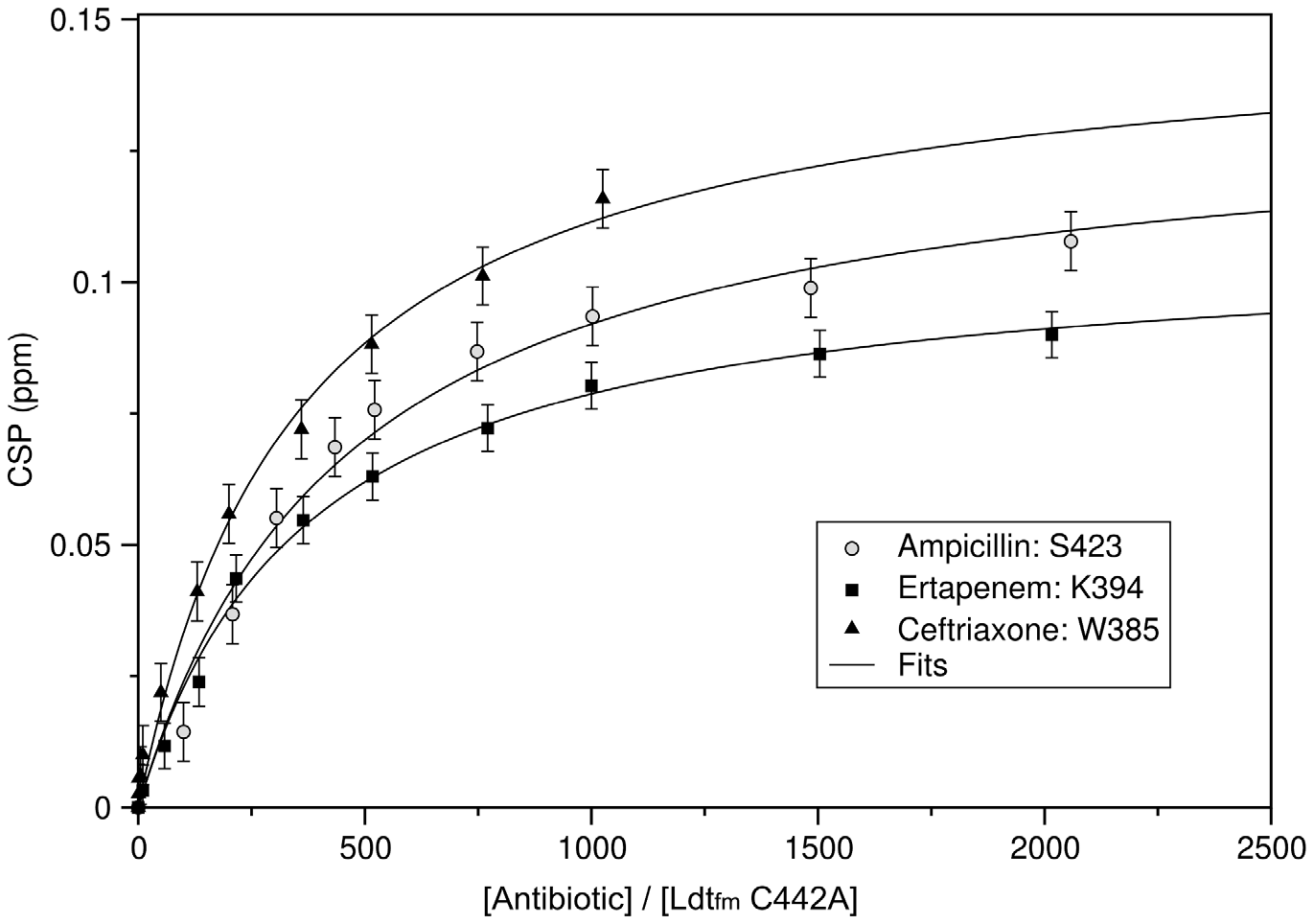
Chemical shift perturbations induced by non-covalent binding of β-lactams to Ldt_fm_ C442A. Chemical shift perturbations (CSPs) of Ldt_fm_C442A residues are reported as a function of the antibiotic to protein molar ratio. Closed square, Lys394 for ertapenem; closed triangle, Trp385 for ceftriaxone; grey circle, Ser423 for ampicillin. The end point of the titration was determined by the solubility limit of the antibiotics. Experimental data were fitted (solid lines) with equation 2 described in the experimental procedures. *K*_D_ values of 50, 44, and 79, mM were determined for binding of ertapenem, ceftriaxone, and ampicillin to Ldt_fm_C442A, respectively. Ertapenem was used as a representative of the carbapenem family since the low solubility of imipenem precluded *K*_D_ determination for this antibiotic.

**Figure 2:**
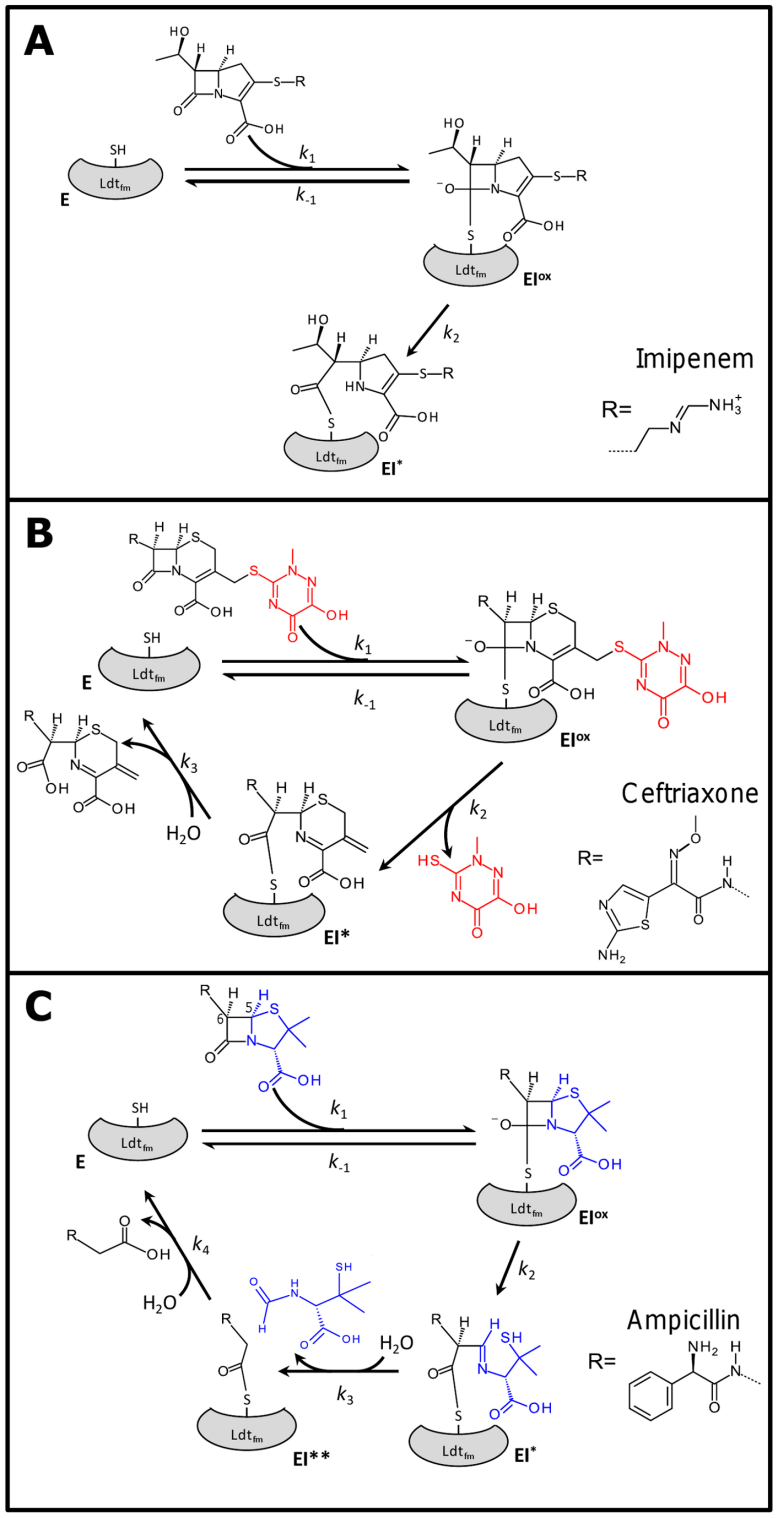
Inactivation of *E. faecium* L,D-transpeptidase (Ldt_fm_) by β-lactams. Reaction schemes for Ldt_fm_ inactivation by β-lactams of the carbapenem (imipenem), cephem (ceftriaxone), and penam (ampicillin) classes. E, free form of the enzyme; EI^ox^, oxyanion; EI* and EI**, acylenzymes. SH, sulfhydryl of the catalytic cysteine.

In order to evaluate non-covalent binding of the drugs to Ldt_fm_, we blocked the acylation step of the reaction by replacing the catalytic cysteine by alanine. The Ldt_fm_ variant harboring the C442A substitution was totally inert when incubated with imipenem, ceftriaxone, and ampicillin as formation of covalent adducts was not detected by mass spectrometry, fluorescence quenching was not observed, and β-lactam hydrolysis was not detected by spectrophotometry (data not shown). Each of these assays is limited with respect to highest drug concentration that is experimentally accessible (in the order of 1 mM, see below). Higher drug concentrations were investigated by multidimensional NMR spectroscopy. The C442A substitution did not alter the chemical shifts of the backbone resonances observed in the [^1^H,^15^N]-HSQC spectrum (data not shown) indicating that the substitution did not modify the protein conformation. NMR chemical shift perturbation experiments were performed by incubating Ldt_fm_C442A with increasing β-lactam concentrations up to the drug solubility limit in order to observe formation of non-covalent complexes (Fig. 1). A fast exchange regime was detected between free enzyme and the complexes. The residues that were affected by drug binding were mostly located at the surface of the protein in the vicinity of the Ldt_fm_C442A catalytic cavity indicating specific binding of the drugs (Supplementary Fig. S1 in File S1). Titration of [^1^H,^15^N]-chemical shift perturbations revealed *K*_D_ values of 50, 44, and 79 mM for binding of ertapenem, ceftriaxone, and ampicillin, respectively. Ertapenem was used instead of imipenem since the lower solubility of the latter carbapenem precluded *K*_D_ determination. These results indicate that differences in the affinity of Ldt_fm_ for β-lactams cannot account for differences in the antibacterial activity of carbapenems, cephems, and penams since representatives of these three β-lactam classes bound to Ldt_fm_C442A with similar low affinities. These results also indicate that non-covalent complexes do not significantly accumulate at the >10^2^-fold lower drug concentrations used in kinetic studies (below). Consequently, reversible formation of tetrahedral oxyanions (EI^ox^), resulting from the nucleophilic attack of the β-lactam ring carbonyl by the active site cysteine thiolate, was considered as the first relevant intermediate in the inactivation reactions depicted in Fig. 2.

### Acylation of Ldt_fm_ by β-lactams Results in the Formation of Various Adducts

Acylation of Ldt_fm_ by imipenem, ceftriaxone, and ampicillin was investigated by mass spectrometry (Table 1) and the deduced reaction schemes are presented in Fig. 2. As previously described [10], incubation of Ldt_fm_ with imipenem resulted in the formation of a single adduct, EI*, with a mass increment corresponding to the mass of the antibiotic. This adduct is generated by formation of a thioester bond between the sulfhydryl group of the Ldt_fm_ active-site cysteine and the carbonyl group of the imipenem β-lactam ring (Fig. 2A) [10]. The mass of the acylenzyme obtained with ceftriaxone (EI*) indicated that a portion of one of the two drug side chains was lost upon acylation (Table 1 and Fig. 2B). Acylation of the active-site cysteine and loss of the drug side chain may occur in a single step since evidence for formation of the complete acylenzyme was not obtained. The same inactivation scheme was recently reported for inactivation of L,D-transpeptidase Ldt_Mt1_ from *M*. *tuberculosis* [13]. Ldt_fm_ formed two acylenzymes with ampicillin (EI* and EI**; Fig. 2C). The mass of EI* corresponds to the complete acylenzyme as found for imipenem. The second acylenzyme, EI**, was generated by additional cleavage of the C^5^–C^6^ bond of the β-lactam ring (Fig. 2C). Thus, ampicillin covalently binds to Ldt_fm_ although this does not lead to antibacterial activity.

**Table 1:** Average mass of acylenzymes (mass increment)^a^.

β-lactam (average mass)	EI*	EI**
Imipenem (299.4)	29,309.7 (300.4)	NA
Ceftriaxone (554.6)	29,406.2 (396.9)	NA
Ampicillin (349.4)	29,359.2 (349.9)	29,200.6 (191.3)

a The mass increment was calculated by subtracting the mass of the native enzyme (29,009.3) from the mass of acylenzymes.

NA, not applicable.

### Partial Acylation of Ldt_fm_ by Ceftriaxone and Ampicillin

**Figure 3:**
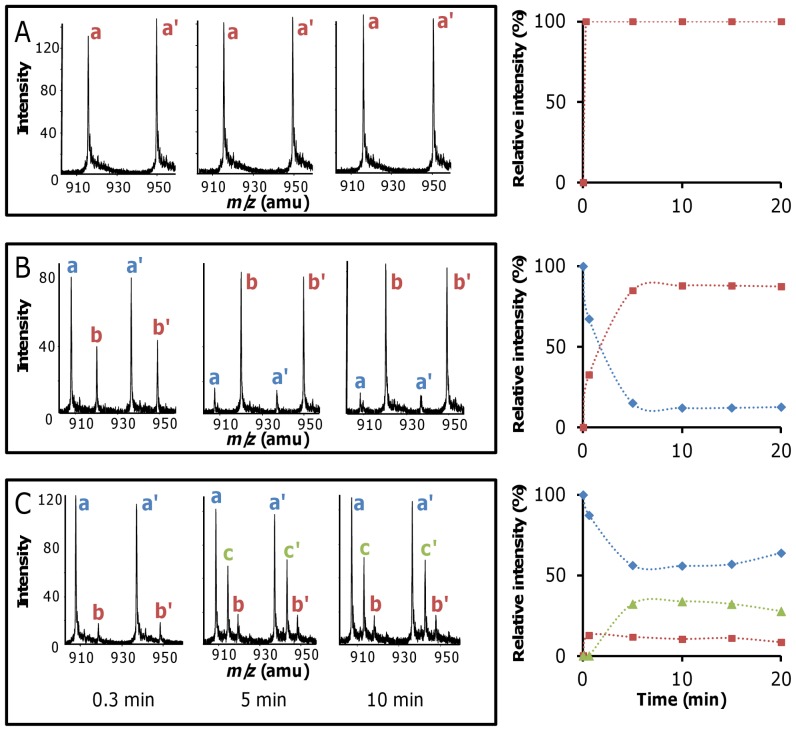
Mass spectrometry analysis of kinetics of Ldt_fm_ inactivation by β-lactams. Ldt_fm_ (20 µM) was incubated with 200 µM of β-lactams. Left panels, representative mass spectra obtained after 0.3, 5, and 10 min of incubation of Ldt_fm_ with indicated β-lactams. Pair of peaks labeled with the same letter are [M+32H]^32+^ and [M+31H]^31+^ ions. (A) imipenem, peaks a and a’ at *m*/*z* 916.93 and 946.48 correspond to acylenzyme EI*. (B) Ceftriaxone, peaks a and a’ at *m*/*z* 907.58 and 936.84 correspond to free enzyme. Peaks b and b’ at *m*/*z* 919.95 and 946.60 correspond to acylenzyme EI*. (C) Ampicillin, peaks a and a’ (*m*/*z* 907.52 and 936.79), b and b’ (*m*/*z* 918.52 and 948.08), and c and c’ (*m*/*z* 913.51 and 942.95) correspond to free enzyme, EI*, and EI**, respectively. Right panels, kinetics of Ldt_fm_-β-lactam adducts formation. Relative intensities were deduced from peak heights. Blue diamond, free enzyme; Red square EI*; Green triangle EI**.

Kinetics of Ldt_fm_ acylation were analyzed by mass spectrometry (Fig. 3). Acylation of Ldt_fm_ by imipenem was too rapid to be kinetically analyzed as acylenzyme EI* was the only Ldt_fm_ detectable form after 0.3 min of incubation (Fig. 3A). Acylation of Ldt_fm_ by ceftriaxone was slower (Fig. 3B). After 5 min, acylenzyme EI* reached a maximum, ca. 88% of total enzyme based on peak height, and this percentage remained stable between 5 and 20 min. The mass deduced from the remaining peaks (12%) corresponded to the mass of the apoenzyme. These peaks may originate from the presence of both free enzyme (E) and the oxyanion (EI^ox^) in the reaction mixture since EI^ox^ may dissociate upon injection in the mass spectrometer and electrospray ionization. For ampicillin, the relative abundance of acylenzymes EI* and EI** reached equilibrium at 5 min (Fig. 3C). The two acylated forms accounted for *ca*. 56% of total enzyme. Kinetics indicated that EI* is an intermediate in the formation of EI**, as indicated in Fig. 2C, since the two enzyme forms accumulated sequentially.

**Figure 4:**
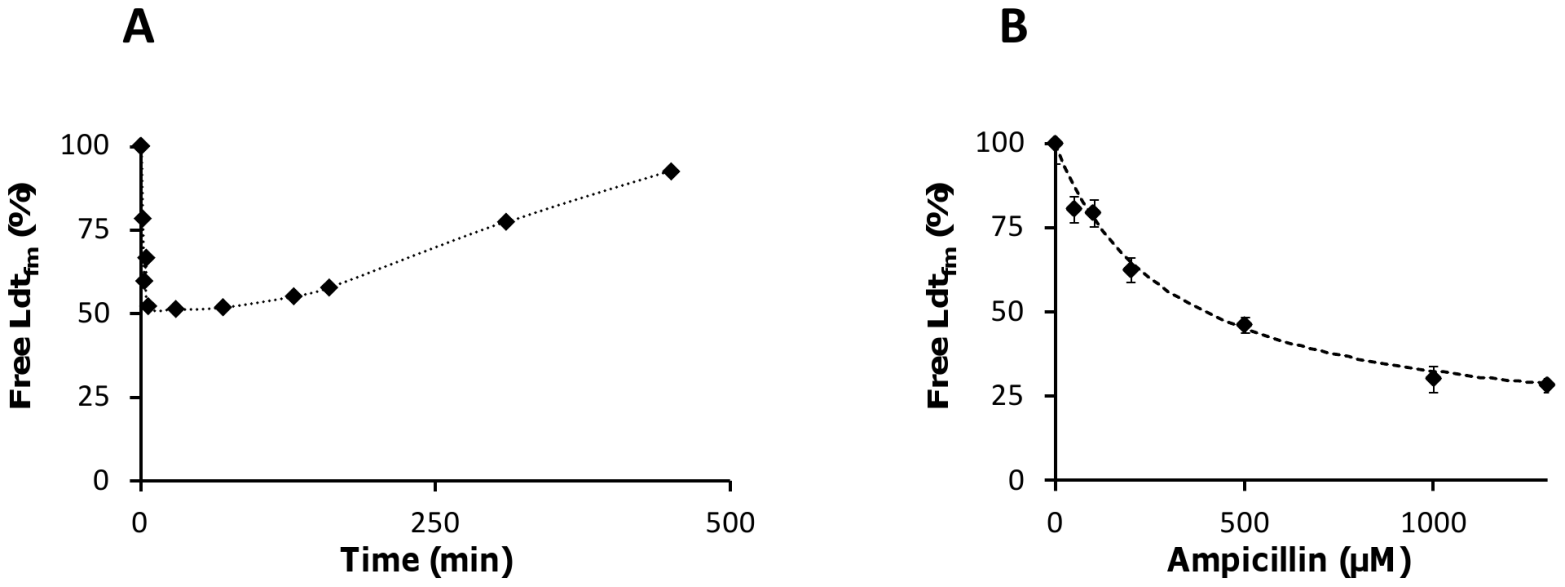
Determination of ampicillin-free Ldt_fm_ using rapid inactivation by imipenem. (A) Ldt_fm_ (20 µM) was incubated with ampicillin (200 µM) for indicated time and imipenem was used to determine the concentration of free enzyme by using stopped-flow spectrophotometry at 299 nm. The concentration of free Ldt_fm_ reached a plateau revealing equilibrium between the various enzyme forms. The concentration of free Ldt_fm_ slowly increased after 100 min due to a decrease in ampicillin concentration. (B) Concentration of free Ldt_fm_ at equilibrium as a function of ampicillin concentration. Data are mean ±SD of 3 experiments.

A second assay was developed to independently evaluate the extent of Ldt_fm_ acylation by ampicillin. The assay relies on rapid acylation of the Ldt_fm_ free form by imipenem, which results in an absorbance decrease at 299 nm due to rupture of the carbapenem β-lactam ring. Ldt_fm_ (20 µM) was incubated with ampicillin (200 µM) and the enzyme free form was titrated at various time intervals (0 to 500 min) based on addition of imipenem to reaction samples. The concentration of free Ldt_fm_ decreased during the first 6 min of incubation with ampicillin to reach 52% of total enzyme (Fig. 4A). Thereafter, the concentration of free enzyme remained stable for 100 min indicating that the different enzyme forms were in equilibrium. The concentration of free Ldt_fm_ at equilibrium was determined for various ampicillin concentrations using the same approach (Fig. 4B). Extrapolation indicated that the abundance of the acylated forms (EI* plus EI**) reached 88% of total enzyme at a saturating concentration of ampicillin. Of note, similar extents of Ldt_fm_ acylation by ampicillin at 200 µM were observed by mass spectrometry (Fig. 3C) and titration with imipenem (Fig. 4A) (44% versus 48%, respectively). Thus, both assays indicated that a substantial portion of Ldt_fm_ remained unacylated at high ampicillin concentrations.

### Acylenzyme Hydrolysis Accounts for Partial Acylation of Ldt_fm_ by Ceftriaxone and Ampicillin

**Figure 5:**
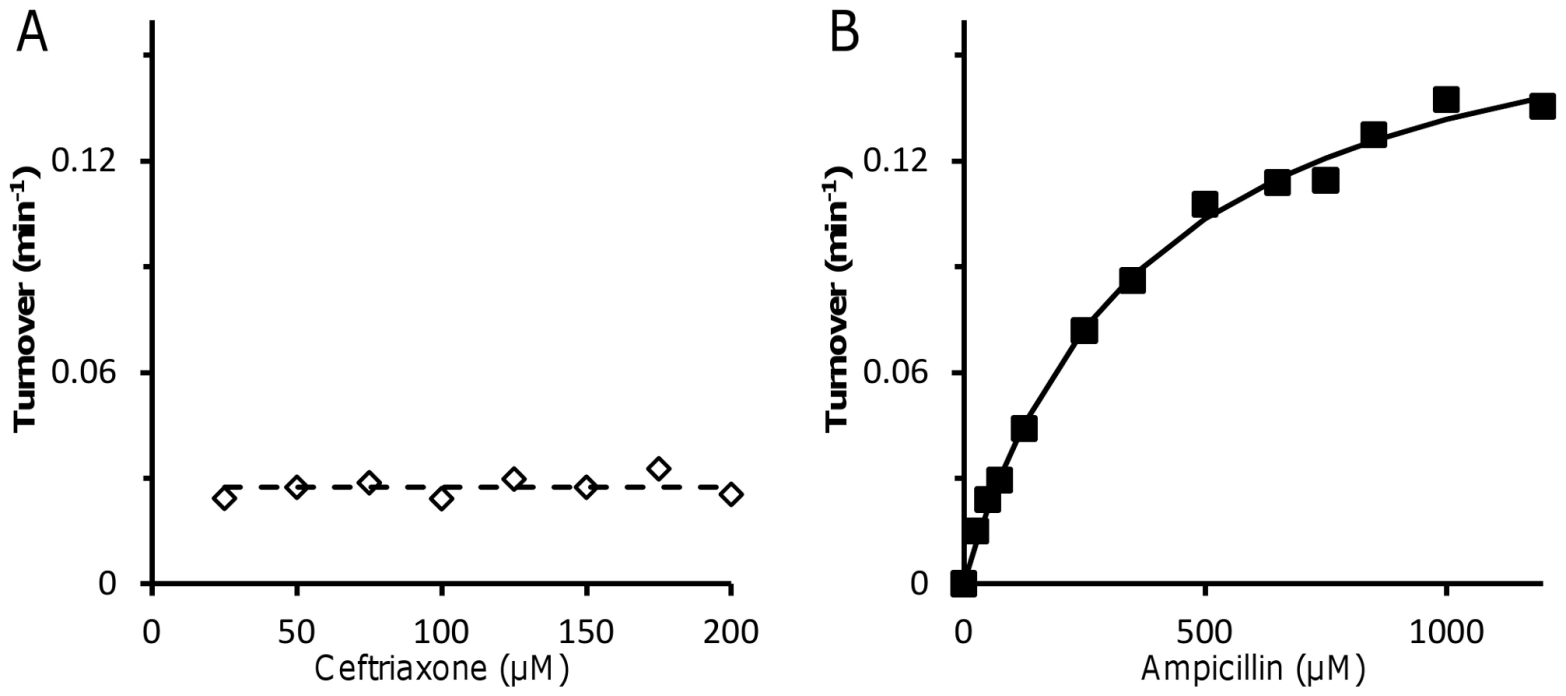
Determination of turnover numbers for full catalytic cycles leading to hydrolysis of β-lactams by Ldt_fm_. Turnover numbers were determined for hydrolysis of ceftriaxone (A) and ampicillin (B) by Ldt_fm_ (5 µM).

Acylenzyme stability was evaluated by determining the rate of hydrolysis of imipenem, ceftriaxone, and ampicillin by Ldt_fm_ (Fig. 5). Enzyme turnover was not detected with imipenem (<4×10^−4^ min^−1^) indicating that the acylenzyme formed with this drug is stable. Ceftriaxone was slowly hydrolyzed by Ldt_fm_ and the turnover number did not vary with the drug concentration (0.027±0.003 min^−1^). The rate of ampicillin hydrolysis increased with the drug concentration in the 25 to 1,200 µM range. The maximum turnover number was 0.18±0.01 min^−1^ and half of this value was reached at an ampicillin concentration of 370±30 µM. The turnover number was higher for ampicillin than for imipenem and ceftriaxone, at least 450 and 6.7 fold, respectively, if a saturating concentration of ampicillin is considered for the comparisons. These results indicate that acylenzyme hydrolysis accounts for the partial acylation of Ldt_fm_ detected by mass spectrometry (Fig. 3) and titration with imipenem (Fig. 4). Equal rates of Ldt_fm_ acylation and acylenzyme hydrolysis lead to equilibrium between the various enzyme forms, which may include sufficient active L,D-transpeptidase to prevent inhibition of peptidoglycan cross-linking by ceftriaxone and ampicillin in vivo.

### Slow Acylation of Ldt_fm_ by Ceftriaxone and Ampicillin Contributes to Partial Enzyme Inactivation

**Figure 6:**
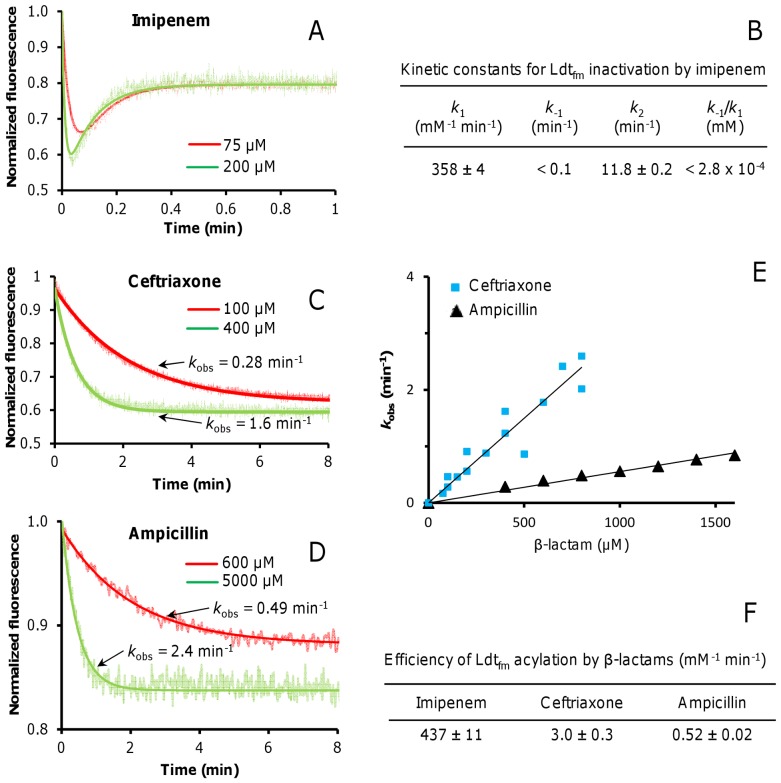
Kinetics of Ldt_fm_ inactivation by imipenem, ceftriaxone, and ampicillin. Fluorescence kinetic data were acquired with a stopped-flow apparatus. Trp residues of Ldt_fm_ were excited at 224 nm and fluorescence emission was determined at 335 nm to monitor quenching upon β-lactam binding. Kinetics were biphasic for imipenem (A) providing estimates of catalytic constants *k*_1_, *k*_−1_, and *k*_2_(B). See Supplementary methods in File S1 for the iterative fitting method and Supplementary Fig. S2 in File S1 for the complete set of data. Monophasic fluorescence decreases observed for ceftriaxone (C) and ampicillin (D) were fitted to exponential decays (representative plots are shown). Regression analysis was performed with equation F_t_ = F_eq_+ΔF e^−*k*obst^ in which F_eq_ and F_t_ are the fluorescence intensities at equilibrium and at time t, respectively, ΔF is the difference between fluorescence intensity at time = 0 and at equilibrium, t is time, and *k*_obs_ is a constant. The resulting rate constants (*k*_obs_) increased linearly with the drug concentration (E) and the slope provided an estimate of the efficiency of enzyme acylation (F).

Ldt_fm_ inactivation by β-lactams was investigated by stopped-flow fluorescence spectroscopy as previously described [12]. Progress curves obtained with imipenem were biphasic (Fig. 6A). The initial rapid fluorescence quenching of Ldt_fm_ Trp residues results from reversible binding of the drug to the enzyme. Formation of the acylenzyme subsequently leads to a fluorescence increase as quenching is less important for EI* than for EI^ox^. This biphasic behavior was used to determine the association rate constant *k*_1_ (359±4 mM^−1^ min^−1^) and the rate constant of the chemical step of the reaction *k*_2_ (11.8±0.1 min^−1^), whereas *k*_−1_ was too low (<0.1 min^−1^) to be determined (Fig. 6B). These values, obtained at 20°C, were slightly higher than those previously reported for assays performed at 10°C [12] (*k*_1_ = 65 mM^−1^ min^−1^; *k*_2_ = 4.5 min^−1^; *k*_−1_<0.1 min^−1^).

Since kinetics of fluorescence quenching were monophasic for ceftriaxone (Fig. 6C) and ampicillin (Fig. 6D), rate constants for binding (*k*_1_) and inactivation (*k*_2_) could not be determined. Fluorescence decreases were fitted to exponential decays and the resulting rate constants (*k*_obs_) were determined for various drug concentrations (Fig. 6E). The rate constant *k*_obs_ increased linearly with the drug concentration and the slope was used as an estimate of the overall efficiency of the acylation reaction (Fig. 6F), as previously described [13]. Based on these estimates, ceftriaxone and ampicillin acylated Ldt_fm_ 150 and 840 fold less efficiently than imipenem.

## Discussion

We have previously showed that activation of a cryptic locus encoding a D,D-carboxypeptidase is the key event that results in activation of the L,D-transpeptidation pathway and high-level resistance to ampicillin in *E*. *faecium* [14]. The D,D-carboxypeptidase generates the tetrapeptide substrate of the L,D-tanspeptidase resulting in mutants that rely exclusively on Ldt_fm_ for peptidoglycan cross-linking [5], [10]. Bypass of PBPs by Ldt_fm_ results in high-level resistance to ampicillin (MIC >2,000 µg/ml) and moderate resistance to ceftriaxone (MIC = 128 µg/ml) whereas imipenem remains active (MIC = 0.5 µg/ml) [10]. The latter drug was found to inactivate Ldt_fm_ in vitro and to block peptidoglycan cross-linking by this enzyme in vivo [10]. Here we show that the three drugs acylate Ldt_fm_ in vitro (Fig. 3) despite the difference in antibacterial activity.

Two approaches identified partial Ldt_fm_ inactivation at all ampicillin concentrations as the basis for the lack of antibacterial activity. First, mass spectrometry indicated that only one half of the enzyme was acylated by ampicillin at 200 µM, a proportion that remained stable upon prolonged incubation (Fig. 3). Second, partial inactivation was detected using an independent assay based on determination of the proportion of Ldt_fm_ that remained able to react with imipenem (Fig. 4). Even at high ampicillin concentration, Ldt_fm_ inactivation was incomplete leading to the persistence of functional enzyme, peptidoglycan cross linking, and high-level drug resistance. The lack of full enzyme inactivation resulted from a combination of acylenzyme instability (Fig. 5) and slow enzyme acylation (Fig. 6). For ceftriaxone, higher acylenzyme stability and acylation rate account for residual activity. Conversely, the excellent antibacterial activity of imipenem results both from the absence of detectable acylenzyme hydrolysis and efficient acylation.

Multidimensional NMR spectroscopy revealed similar high *K*_D_ values for non-covalent binding of ertapenem (50 mM), ceftriaxone (44 mM), and ampicillin (79 mM) to Ldt_fm_C442A, which cannot be acylated (Fig. 1). These results indicate that the substrate specificity of Ldt_fm_ for β-lactams is not determined at a non-covalent binding step of the reaction that would precede nucleophilic attack of the β–lactam carbonyl by the active-site cysteine. The ceftriaxone and ampicillin concentrations for half saturation of the β-lactamase activity of Ldt_fm_ were <0.025 and 0.37±0.03 mM, respectively (Fig. 5). These values are 2 to 3 orders of magnitudes lower that the *K*_D_ values determined by NMR for Ldt_fm_C442A (Fig. 1). Thus, incubation of Ldt_fm_ with submilimolar concentrations of β-lactams, as used for kinetic analyses, cannot result in significant accumulation of a non-covalent complex. The saturable step observed in kinetic analyses of β-lactamase activity was therefore assigned to reversible formation of the oxyanion (Fig. 2).

In order to identify the limiting step in acylation of Ldt_fm_ by ampicillin, data generated by mass spectrometry (Fig. 3) and titration with imipenem (Fig. 4) were combined to roughly estimate the relative concentrations of the four enzyme forms (E, EI^ox^, EI*, and EI**). Free Ldt_fm_ detected by these methods may correspond to E and EI^ox^ since formation of the oxyanion is reversible. At a saturating ampicillin concentration, the velocity of β-lactam hydrolysis is maximal (Fig. 5), and the concentration of E tends to zero. Under such conditions, ca. 12% of the enzyme was accessible to titration by imipenem (Fig. 4) providing an estimate for the oxyanion. Since the relative abundance of EI* and EI** does not vary and can be deduced from MS analyses (30% versus 70%, Fig. 3C), the proportions of E, EI^ox^, EI*, and EI** at a saturating ampicillin concentration can be estimated to be 0, 12, 26, and 62%, respectively. The maximum turnover for the β-lactamase activity, 0.18±0.01 min^−1^, enables to deduce the values of *k*_2_, *k*_3_, and *k*_4_ from these values (1.5, 0.69, and 0.29 min^−1^, respectively). At a concentration of ampicillin of 370 µM, corresponding to the drug concentration required to reach half of the maximum hydrolysis velocity (Fig. 5), the relative abundance of E, EI^ox^, EI*, and EI** can be estimated to be 50, 6, 13, and 31%, respectively, since half of Ldt_fm_ does not participate in ampicillin hydrolysis (relative proportion of E = 50%) and the relative abundance of the three remaining enzyme forms (EI^ox^, EI*, and EI**) do not vary according to the reaction scheme depicted in Fig. 2C. From these values, the dissociation constant of the oxyanion is 120 µM^−1^ as *k*_−1_ EI^ox^ = *k*_1_[E][I] at equilibrium. This value is at least 430 fold higher than that obtained for imipenem (<0.28 µM; Fig. 5B). In contrast, the rate constant for the acylation step, *k*_2_ is only 8 fold lower than that observed for imipenem (1.5 versus 11.8 min^−1^; Fig. 6B). Thus, 840-fold higher efficiency of Ldt_fm_ acylation by imipenem in comparison to ampicillin is predominantly due to oxyanion stabilization. Of note, a low binding affinity for carbapenems was also reported for a C354A mutant of Ldt_Mt2_ from *M. tuberculosis* indicating that this conclusion may be extended to other members of the L,D-transpeptidase family [15].

The factors that contribute to the stability of acylenzymes are unknown. Incubation of Ldt_fm_ with ampicillin resulted in sequential formation of acylenzymes EI* and EI**, as acylation of the active site cysteine was followed by rupture of the β-lactam C^5^–C^6^ bond (Fig. 2C). Sequential formation of the two acylenzymes is supported by the nearly exclusive presence of EI* after 0.3 min of incubation and by detection of the hydrolysis product of EI** but not that of EI* (monoisotopic mass of 208.08 and 367.12, respectively). Rupture of the C^5^–C^6^ bond was previously reported for acylation of R61 D,D-carboxypeptidase by ampicillin [16] but the corresponding fragment is not formed upon catalytic hydrolysis of penams by β-lactamases [17]. Thus, rupture of the C^5^–C^6^ is catalyzed by Ldt_fm_ as previously concluded for R61. Detection of the hydrolysis product of EI** but not that of EI* indicates that hydrolysis of the thioester bond is favored by rupture of the C^5^–C^6^ bond. Thus, this Ldt_fm_-catalyzed reaction is identified here as a factor that destabilizes the acylenzyme and contributes to the lack of antibacterial activity of ampicillin.

L,D-transpeptidases have been recently recognized as the main cross-linking enzymes in *M*. *tuberculosis* [6]. As Ldt_fm_ from *E*. *faecium*, these enzymes are efficiently inactivated by carbapenems [13]. These observations combined to the fact that clavulanic acid irreversibly inactivates the *M*. *tuberculosis* β-lactamase [18], [19] have raised considerable interest in using carbapenems for treatment of extensively drug resistant tuberculosis [19]–[21]. Development of an oral drug is needed for tuberculosis therapy as approved carbapenems are only administrable by the parenteral route, which is not broadly applicable in clinical settings in which extensively drug-resistant tuberculosis is prevalent [20], [22]. Our analysis has revealed key features of efficient β-lactams that will be critical for drug development, namely oxyanion stabilization, which was identified as the limiting step for efficient acylation, and absence of acylenzyme hydrolysis, which was identified as essential for full enzyme inactivation. The former feature mainly depends upon the reactivity of the β-lactam ring, which appears to be determined by the conjugated ring rather than by the drug side chains, as previously concluded from NMR-based analyses of the structure and dynamics of the model L,D-transpeptidase from *Bacillus subtilis* [23] and *Enterococcus faecium* [24]. The latter feature is modulated by secondary catalytic reactions that lead to elimination of a portion of the drug molecules prior to hydrolysis of the thioester bond. These kinetic analyses in combination with recent determination of the structures of *E*. *faecium* Ldt_fm_ [24] and *M*. *tuberculosis* Ldt_Mt2_ [25], [26] acylated by a carbapenem or in complex with a peptidoglycan fragment [15] will pave the way for optimization of the β-lactam scaffold.

## Supporting Information

File S1 (DOCX)
