# Supplementary material for: Kinetic Features of L,D-Transpeptidase Inactivation Critical for β-Lactam Antibacterial Activity
Source: PLoS One. 2013 Jul 4;8(7):e67831. doi: 10.1371/journal.pone.0067831 (PMC3701632; doi:10.1371/journal.pone.0067831)
Supplement: File S1 — (DOCX) [file pone.0067831.s001.docx]

**Kinetic features of L,D-transpeptidase inactivation critical for β-lactam antibacterial activity***

**Sébastien Triboulet^1,2,3¶^, Vincent Dubée^1,2,3¶^, Lauriane Lecoq^4,5,6^, Catherine Bougault^4,5,6^, Jean-Luc Mainardi^1,2,3,7^, Louis B. Rice^8^, Mélanie Ethève-Quelquejeu^9,10^, Laurent Gutmann^1,2,3,7^, Arul Marie^11,12^, Lionel Dubost^11,12^, Jean-Emmanuel Hugonnet^1,2,3^, Jean-Pierre Simorre^4,5,6^, and Michel Arthur^1,2,3^**

^1^Centre de Recherche des Cordeliers, Equipe 12, Université Pierre et Marie Curie–Paris 6, UMR S 872, Paris, France

^2^INSERM, U872, Paris, France

^3^Université Paris Descartes, Sorbonne Paris Cité, UMR S 872, Paris, France

^4^ CEA, DSV, Institut de Biologie Structurale (IBS), Grenoble, France

^5^CNRS, UMR 5075, Grenoble, France

^6^Université Joseph Fourier, Grenoble 1, France

^7^Assistance Publique-Hôpitaux de Paris, Hôpital Européen Georges Pompidou, Paris, France

^8^Rhode Island Hospital, Brown University, Providence, Rhode Island

^9^Laboratoire de Chimie et de Biochimie pharmacologiques et toxicologiques, Université Paris Descartes, UMR 8601, Paris, France

^10^CNRS, UMR 8601, Paris, France

^11^Muséum National d’Histoire Naturelle, USM0502, Plateforme de Spectrométrie de Masse et de Protéomique du Muséum, Paris, France

^12^CNRS, UMR8041, Paris, France

*Running title: L,D-transpeptidase inactivation by β-lactams

To whom correspondence should be addressed: Michel Arthur, Centre de Recherche des Cordeliers, Equipe 12, Université Pierre et Marie Curie–Paris 6, 15 rue de l’Ecole de Médecine, Paris, F-75006 France, Tel.: (33) 1 44 27 54 55; Fax: (33) 1 44 27 23 36; E-mail: michel.arthur@crc.jussieu.fr. Jean-Pierre Simorre, Institut de Biologie Structurale Jean-Pierre Ebel, 41, avenue Jules Horowitz, 38000 Grenoble, France, Tel.: (33) 4 38 78 57 99; Fax: (33) 4 38 78 54 94; E-mail: jean-pierre.simorre@ibs.fr

**^¶^**Both authors contributed equally to this work

**Keywords:** Extensively drug-resistant tuberculosis; L,D-transpeptidase; β-lactam resistance

**SUPPLEMENTARY METHODS**

*Determination of kinetic constants for inactivation of Ldt_fm_ by imipenem–*Ldt_fm_ (5 µM) was incubated with various imipenem concentrations (25 to 200 µM) at 20 °C in 100 mM sodium phosphate (pH 6.0) (Supplementary Fig. S2A). Fluorescence kinetics were performed with a stopped-flow apparatus (RX-2000, Applied Biophysics) coupled to a spectrofluorometer (Cary Eclipse; Varian) (λ_ex_ 224 nm, optical path length of 2 mm; λ_em_ 335 nm, 10 mm) with slits of 5 nm. Kinetic constants *k*_1_, *k*_-1_, and *k*_2_, were determined as previously described [1]. Briefly, variations in the concentrations of the three forms of Ldt_fm_ over time were defined for free enzyme (d[E]/dt = *k*_-1_[EI^ox^] – *k*_1_[E][I], which is equal to d[I]/dt), for the oxyanion (d[EI^ox^]/dt = *k*_1_[E][I] – *k*_­1_[EI^ox^] – *k*_2_[EI^ox^]), and for the acylenzyme (d[EI*]/dt = *k*_2_[EI^ox^]). Fluorescence intensity (F) was considered to be the sum of the relative fluorescence intensities of the three forms of the enzyme. Progress curves were fitted with Excel (Microsoft®) as previously described [1]. Determination of the values of kinetic constants *k*_1_, *k*_-1_, and *k*_2_, and statistical analysis were performed with Dynafit Software (Biokin, Watertown, MA) [2].

In order to compare the efficacy of Ldt_fm_ inactivation by imipenem, ceftriaxone, and ampicillin the values of *k*_1_, *k*_-1_, and *k*_2_ were used to determine the *k*_inact_ / *K*_app_ ratio, as previously described [3]. Briefly, the sum of the concentrations of [E] and [EI^ox^] was calculated and plotted as a function of time for various imipenem concentrations and [E_total_] = 1 µM (supplementary Fig. S2B). Kinetic constant *k*_obs_ was obtained for each concentration by fitting simulations to equation [E_total_] - [EI*] = [E_total_]e*^-k^*^obst^, in which [E_total_] is the total enzyme concentration, *k*_obs_ a constant, and t time (1). *k*_obs_ values were plotted as a function of imipenem concentrations and regression analysis (Supplementary Fig. S2C) was performed to determine constants *k*_inact_ and *K*_app_ according to equation *k*_obs_ = *k*_inact_[I] / (*K*_app_ + [I]), in which *k*_inact_ is the first-order constant for acylenzyme formation and *K*_app_ a constant. The *k*_inact_ over *K*_app_ ratio was used as an estimate of the efficiency of Ldt_fm_ inactivation.

S**UPPLEMENTARY FIGURE LEGENDS**

**SUPPLEMENTARY FIGURE S1. Chemical shift perturbations induced by non-covalent binding of ampicillin, ertapenem and ceftriaxone on Ldt_fm_ C442A.** ^1^H,^15^N-chemical shift perturbations (calculated using equation (1) in the experimental procedures) induced by addition of (A) 525 equivalents of ampicillin, (B) 517 equivalents of ertapenem and (C) 515 equivalents of ceftriaxone in Ldt_fm_ C442A (150 µM) are represented as a function of the protein primary sequence. Residues showing medium CSPs (between 0.03 ppm and 0.1 ppm) and strong CSPs (greater than 0.1 ppm) are colored on the X-ray structure of Ldt_fm_ in orange and red, respectively, for (D) ampicillin, (E) ertapenem and (F) ceftriaxone. Both views of the front and the back of the protein are provided. Residue at position 442 (in yellow) was also significantly affected along the titrations with ampicillin and ertapenem. Prolines are shown in grey on the structure. (G) Cartoon representation showing structural elements of Ldt_fm_.

**SUPPLEMENTARY FIGURE S2. Kinetics of Ldt_fm_ inactivation by imipenem.** (A) Fluorescence kinetics obtained with four concentrations of imipenem (blue curves). Solid lines correspond to fits. (B) The sum of the concentrations of the enzyme free form [E] and of the oxyanion [EI^ox^] were calculated using kinetic constants *k*_1_, *k*_-1_, and *k*_2_ (light blue). Fits to exponential decay was used to deduce *k*_obs_ from each plot. (C) *k*_obs_ values were plotted as a function of imipenem concentration to determine constants *k*_inact_ and *K*_app_.

**SUPPLEMENTARY REFERENCES**

1 Triboulet S, Arthur M, Mainardi JL, Veckerle C, Dubee V, et al. (2011) Inactivation kinetics of a new target of beta-lactam antibiotics. J Biol Chem 286: 22777–22784.

2 Kuzmic P (1996) Program DYNAFIT for the analysis of enzyme kinetic data: application to HIV proteinase. Anal Biochem 237, 260-273

3 Dubée V, Triboulet S, Mainardi JL, Ethève-Quelquejeu M, Marie A, et al. (2012) Inactivation of *Mycobacterium tuberculosis* L,D-transpeptidase Ldt_Mt1_ by carbapenems and cephalosporins. Antimicrob Agents Chemother 56: 4189–4195.
